# The Methods of Using Argyrol1Read by invitation at the 14th annual meeting of the Tri-state—Alabama, Georgia, Tennessee—Medical Society, Birmingham, Ala., October 9, 1902.

**Published:** 1902-11

**Authors:** A. C. Barnes

**Affiliations:** Philadelphia, Pa., 24 North Fortieth Street.


### The Methods of Using Argyrol.[1]

BY A. C. BARNES, M. D., Philadelphia, Pa.

IN ACCEPTING your kind invitation to read a paper before you, I am deeply conscious of the honor conferred upon me, because many of your members occupy positions equal in honor and eminence with the leaders in modern progressive medicine. The subject I have chosen was selected for two reasons: first, as a body of practical physicians interested in the extremely important question of the treatment of disease, my subject will probably be interesting; second, my paper will be of the nature of an open letter in reply to many inquiries received from physicians in practically every state in the Union.

---

1. Read by invitation at the 14th annual meeting of the Tri-state—Alabama, Georgia, Tennessee—Medical Society, Birmingham, Ala., October 9, 1902.

The original report of my colleague, Dr. Herman Hille, and myself, *Medical Record*, May 24, 1902, concerning our discovery of a new silver salt, was given considerable prominence in the medical press of America and Europe, particularly because of its wide field of application in therapeutics. This salt now known as argyrol is chemically silver vitellin, the principal features of which are the high amount of silver contained, its easy solubility, its intense penetrative action and its freedom from the irritating properties possessed by the other silver salts. It is beyond the scope of this paper to deal with the chemical nature of the salt, and those interested therein are referred to our original report.

It is to the clinical applications of argyrol that I would now direct your attention and more especially to the methods of using the product in inflammatory conditions of the eye, ear, nose, throat and genitourinary organs. The methods herein mentioned are those employed in the various clinics in many hospitals, including the University of Pennsylvania, City Hospitals of New York and Boston, Jefferson, Good Samaritan, Berlin Polyclinic, Children's Hospital, Philadelphia, and in some eye and ear infirmaries of several of our large cities, by surgeons whose names and reputations are well known to you: Martin, Thomson, Horwitz, Swinburne, Christian, Lewis, Lederman, Mellor, etc. Most of these surgeons are preparing or have already finished clinical reports embodying their experiences with the salt which will be published shortly. My paper will be merely a short résumé of the methods of using the product now in vogue.

*Diseases of the Eye.*—Those oculists using argyrol employ it in the conditions formerly treated by silver nitrate or protargol. The rationalé of its use in these diseases is based upon its high proportion of silver, its deep penetrative action and its entire freedom from irritating properties. For instance, a 20 per cent. solution of argyrol corresponds to about a 10 per cent. solution of silver nitrate, yet this strength of argyrol may be dropped in the normal eye without producing irritation or discomfort.

*Purulent Conjunctivitis.*—A 25 per cent. solution has been found to be the proper strength for routine use. Well established cases of ophthalmia neonatorum thus treated will be eradicated in 2 to 3 days. In the last 10 cases of this affection treated by Mellor at the University Hospital, one day's use of a 25 per cent. solution argyrol sufficed to rid the eyes of pus and effect uninterrupted recoveries. The argyrol solution should be dropped in all parts of the conjunctival sac every 3 or 4 hours. With treatment instituted early in the disease corneal complications do not occur.

*Gonorrheal Ophthalmia.*—This is best treated by strengths of 25 to 50 per cent. solutions, according to the stage and extent of the infection. In very severe cases a 50 per cent. solution instillated every 2 or 3 hours produces a reduction of the purulent secretion and affords comparative relief from pain.

An ordinary early case of this disease treated with free use of a 25 per cent. solution every two or three hours will terminate within a few days. For the catarrhal condition of the conjunctiva, resulting from gonorrheal ophthalmia, many oculists direct the instillation of a 10 per cent. solution of argyrol, 3 or 4 times daily; this may be done with perfect safety by the patient at home.

The effects of argyrol in trachoma are still unsettled. Gilfillan, of New York, used it at the House of Refuge with indifferent results. Thomson mentions one very pronounced case in which the lids were so swollen that it resembled ptosis and in which he obtained great improvement by painting the affected lids with a 20 per cent. argyrol solution. This case had been treated with protargol without benefit.

For ordinary catarrhal conjunctivitis a 5 or 10 per cent. solution for use by the patient at home, 3 times daily, with the local application of a few drops of a 25 per cent. solution by the attending physician, produces in most instances prompt and permanent benefit; this same method of treatment is employed in blepharitis, blepharo-conjunctivitis and blennorrhea. The most suitable strength for all around office use in treating corneal ulcers and the ordinary inflammatory conditions of the eye is 25 per cent.; this strength does not cause irritation or discomfort.

The methods of using argyrol in diseases of the *nose*, *throat* and *ear* are perhaps best illustrated by quoting the experience of Dr. M. D. Lederman, of New York, who has been using it for four months in his private work and at his clinics at the Manhattan Eye and Ear Hospital and at the New York Polyclinic. Dr. Lederman states:

I have employed solutions from 10 to 50 per cent. in catarrhal manifestations of the nasal, pharyngeal and laryngeal mucous membrane; the applications were made with the usual cotton carrier every other day. The advantage this silver salt distinctly demonstrates is its freedom from irritation when applied to sensitive mucous membranes. In acute and subacute laryngitis, I have used a 10 per cent. solution increasing to 30 per cent. without the least unpleasantness to the patient. After 2 or 3 treatments the congested appearance of the membrane gradually left and the voice returned in good volume. I particularly noticed that the harsh and dry sensation produced by silver nitrate was never experienced. The secretion was promptly stimulated by the argyrol solutions and produced a comfortable feeling of moisture in the pharynx and larynx. In postnasal catarrh, the character of the discharge was influenced by the argyrol solutions (20, 30 and 50 per cent.) The thick plugs of mucus, so frequently expectorated in cases of nasopharyngitis and in inflammations of the lymphoid tissues in the pharyngeal vault, became more fluid in consistency —showing the stimulating effect of the drug upon the mucous glands—and thus permitted the reëstablishment of the normal function of the membrane and relieved the annoying symptoms of hacking and dropping in the throat; the same effects were noted from applications to the nasal mucous membrane.

The bland nature of the argyrol solutions was especially observed in cases of so-called "Hay Fever." Argyrol, 10 and 20 per cent. solutions, while naturally exciting some sneezing, as would result from any foreign element, seemed to lessen the existing hyperesthesia and retard the excessive flow of secretion; this blennostatic action, I believe, is due to the deep penetration of the argyrol.

The decided anti-germicidal action of the salt is illustrated by its effects in cases of chronic purulent otitis media with osseous necrosis. In these cases I employ a 50 per cent. solution freely in the middle-ear cavity without any annoyance to the patient. The purulent character of the discharge is obviously modified after a few treatments and assumes a mucoid appearance.

In empyema of the antrum of Highmore, Hitschler used a 50 per cent. solution of argyrol once daily and notes prompt disappearance of the purulent discharge.

*Genitourinary Diseases.*—Dr. Orville Horwitz, professor of genitourinary surgery, Jefferson Medical College, treats acute cases of gonorrhea by ordering the hand injection of a 5 per cent. solution of argyrol several times daily with whatever modifications and additions to treatment the cases may demand.

In acute gonorrhea, Dr. H. M. Christian, professor of genitourinary diseases, Philadelphia Polyclinic, employs a 2 to 5 per cent. solution by injection (by ordinary hand syringe) 3 or 4 times daily; the solution is held in the urethra five minutes. If the entire urethra is involved, he employs daily irrigations of 1 to 1000 solution.

In chronic posterior urethritis he makes deep instillations of 5 or 10 per cent. solutions. Of his first 48 acute cases thus treated, 43 showed complete disappearance of gonococci from the discharge within 14 days; 38 of these patients were discharged cured in from 2 to 4 weeks. In no instance did the injections produce irritation or discomfort.

Dr. G. K. Swinburne, surgeon to the Good Samaritan Dispensary,—the largest genitourinary clinic in New York—has treated over 400 cases of gonorrhea with argyrol. His methods are as follows: in acute cases he irrigates the urethra daily with a 1 to 1000 or 1 to 2000 warm argyrol solution and follows this by a 2 to 5 per cent. injection. If the patient cannot report daily he orders the home use of a 2 per cent. injection. He uses argyrol solution for irrigation where formerly he used potassium permanganate or protargol, because of better results and greater comfort to the patient.

In posterior urethritis and cystitis he makes deep instillations of a 5 or 10 per cent. solution. In chronic cases and in those requiring sounds he employs an ointment of 5 per cent. argyrol in lanoline, the ointment being distributed along the urethra by the successive use of several sounds upon the end of each of which the ointment is placed.

In acute cases of gonorrhea seen during the first or second day of the attack, he injects a 20 per cent. solution, and has succeeded in aborting the disease.

Dr. Bransford Lewis, professor of genitourinary surgery, Marion-Sims-Beaumont Medical College, St. Louis, says that he has had much greater success in both aborting and curing gonorrheal infections with argyrol than with any medicament he has ever used. For the abortive treatment of a beginning infection in the male, he prescribes a 2 to 4 per cent. solution for the patient to inject with a No. 23 Goodyear syringe, every two or three hours, day and night, holding it in the urethra four minutes each time, and has the patient call at the office once daily for a pint irrigation of a 2 per cent. solution. If the infection is not so recent, and both parts of the urethra are affected, Dr. Lewis has the patient use both irrigations and anterior injections each day, the former with a one quart fountain syringe, 10 ounces of 1 per cent. solution, and has the patient call at the office every one or two days in order to reinforce medication and to watch progress, or make variations in measures. For the female, in addition to urethral and vaginal irrigations, according to the location of the infection, Dr. Lewis gently cleans the cervical canal and inserts into it a small strip of narrow gauze soaked in 10 per cent. argyrol solution, leaving the end of the strip hanging out so that the woman can remove it on taking her next douche; a pledget of argyrol-soaked cotton is also left in the vagina.

Briefly stated, the advantages noted in argyrol treatment of urethritis are: the shorter the duration of the disease, the power of the drug to allay the inflammation, the comparative comfort afforded the patient and the entire freedom of the injections from irritating properties.

*Diseases of Women.*—In specific urethritis in the female, Kevin injects a 10 per cent. solution into the urethra and bladder. In purulent conditions of the vaginal mucous membrane, the vagina is douched with a 1 to 2000 or 1 to 1000 argyrol solution, after which local applications of a 25 to 50 per cent. solution are made through a speculum; these same methods are employed in ulcerations and erosions of the cervix.

Cases of cystitis are irrigated with 1 to 1000 solution, followed by the injection of a 5 or 10 per cent. solution into the bladder, which is retained there for a few minutes and then discharged by urination.

In obstetrics, argyrol is probably destined to play an important part because of its usefulness as a prophylactic against ophthalmia neonatorum. In several maternity hospitals the instillation of a 1 or 2 per cent. solution into the newly born infant's eyes, is a routine practice.

Other clinical conditions in which the use of argyrol has been suggested and is being tried are erysipelas (suggested by Dr. E. B. Gleason, Medico-Chirurgical Hospital, as local applications 25 to 50 per cent. solution) and certain pathological conditions of the mouth and teeth (suggested by Dr. W. H. Snider, of the University of Buffalo.) It is too soon to make any positive statement of the methods or effects of using argyrol in these two latter conditions.

It will be noted in reviewing my paper that argyrol has been used in almost every branch of surgery, but it will be recalled also that silver has been for many years the principal drug in nearly all of these conditions. Silver nitrate is a very valuable remedy, but its chemical nature necessarily endows it with certain drawbacks, viz.: it is irritating, caustic, is chemically changed by the secretions, and is not penetrating much beyond the surface. Argyrol is not chemically changed by the secretions, possesses intense penetrative power, whereby the effects of silver are exerted in the sub-mucous structures (where they are most needed) and may be used in any structure of the body, in almost any strength without destroying tissue or producing irritation. Furthermore (as all the surgeons mentioned herein have noted and commented upon), argyrol has one very marked property, *i. e.*, its effects in allaying the signs and symptoms of inflammation.

24 NORTH FORTIETH STREET.

